# Draft Genome Sequence of Novel *Metschnikowia* sp. Strain JCM 33374, a Nectar Yeast Isolated from a Bumblebee

**DOI:** 10.1128/MRA.00704-19

**Published:** 2019-09-12

**Authors:** Akira S. Hirao, Ryosuke Imai, Rikiya Endoh, Moriya Ohkuma, Yousuke Degawa

**Affiliations:** a Sugadaira Research Station, Mountain Science Center, University of Tsukuba, Ueda, Nagano, Japan; b Faculty of Symbiotic Systems Science, Fukushima University, Fukushima, Fukushima, Japan; c Iriomote Station, Tropical Biosphere Research Center, University of the Ryukyus, Nishihara, Okinawa, Japan; d Microbe Division, Japan Collection of Microorganisms, RIKEN BioResource Research Center, Tsukuba, Ibaraki, Japan; University of California, Riverside

## Abstract

Here, we report the draft genome sequence of Metschnikowia sp. strain JCM 33374, a nectar yeast isolated from a bumblebee (Bombus diversus). The genome of 20.1 Mb is a naturally heterozygous diploid. Phylogenetic analysis with related taxa demonstrated that the strain is very likely a novel species.

## ANNOUNCEMENT

The genus Metschnikowia (Saccharomycetaceae) consists of more than 80 validly described species of ascomycetous yeasts (1), characterized by the formation of needle-shaped ascospores as their sexual forms (2). *Metschnikowia* species show a high degree of ecological specialization (3), some of which are found at the plant-insect interface (4–7). We isolated *Metschnikowia* sp. strain JCM 33374 from nectar carried by a worker bumblebee (Bombus diversus) (Hymenoptera: Apidae) foraging flowers of red clover Trifolium pratense on a grassland in Sugadaira, Nagano Prefecture, Japan (36.523857°N, 138.348215°E). To retrieve nectar from a crop of the bumblebee, we gently squeezed her abdomen to regurgitate her crop content, which was then collected and stored using a sterile 10-μl microcapillary tube (Drummond Scientific, USA). We cultivated the strain in yeast-malt (YM) liquid medium (Difco, USA) at 20°C for 3 days, and the genomic DNA was extracted using the Genomic-tip 100/G extraction kit (Qiagen, USA). Genome sequencing was performed on the MinION system (Oxford Nanopore Technologies, UK) using an R9.4 flow cell with the rapid sequencing kit (SQK-RAD002) protocol, or on the MiSeq platform (Illumina, USA) using approximately 5% of a flow cell with the 2 × 300-bp paired-end protocol. The MinION system generated 105,151 reads (mean length, 5,514 bp), while the MiSeq platform generated 1,067,271 pairs of reads (mean length, 284 bp) after filtering by fastp v0.19.6 (8), both of which were processed for the following analyses. The combined assembly of the MinION and MiSeq reads was performed following the assembly-polish pipeline (https://github.com/nanoporetech/ont-assembly-polish), which integrated the assembler Canu v1.7 (9) for the long MinION reads with the consensus module Racon v1.3.1 (10) and the assembly improvement tool Pilon v1.22 (11) tailored for polishing based on the short Illumina reads.

The assembled draft genome consisted of 20,114,833 bp from 165 contigs, with a G+C content of 43.5%. The longest contig was 1,028,286 bp long, and the *N*_50_ value was 208,628 bp. Average coverages were 16.4× and 32.3× for the MinION and the MiSeq reads, respectively. This draft genome sequence contains 90.3% (1,473 complete and 72 fragmented) of the 1,711 benchmarking universal single-copy orthologs (BUSCOs) (BUSCO v3.0.2 [12]) using the OrthoDB v9 data set for Saccharomycetales. Protein-coding genes were annotated using AUGUSTUS v3.3.1 (13) with a Saccharomyces cerevisiae training set, which predicted 6,685 genes. tRNAscan-SE v2.0 (14) identified 240 tRNA genes. Genome Analysis Tool Kit (GATK v4.1.2.0) (15) best practices were performed to filter and accept a total of 148,417 single nucleotide polymorphisms (SNPs) and 30,795 insertion and deletions (indels). The observed distributions of allele depth ratios for all SNPs and indels exhibited a single peak at around 0.5, representing that the naturally heterozygous genome is diploid.

Phylogenetic analysis was performed to determine if the strain could be part of a novel species. A maximum likelihood tree was constructed using RAxML v8 (16) on the sequence of the D1/D2 domain of the large-subunit (LSU) rRNA gene from the draft genome with those from the *Metschnikowia* clade available in the DDBJ/ENA/GenBank database. The phylogenetic tree revealed that the strain located in the *Metschnikowia* clade but was different from any described *Metschnikowia* species. The sequence of the LSU D1/D2 from the strain was validated by Sanger sequencing and showed 6.3% divergence from that of the closely related species Metschnikowia lachancei (17). For ascomycetous yeasts, more than 1% divergence in LSU D1/D2 represents the threshold used to discriminate a species (18). Therefore, the strain is very likely a novel species.

### Data availability.

The draft genome sequence and annotation data of *Metschnikowia* sp. strain JCM 33374 have been deposited at DDBJ/ENA/GenBank under the accession number BIMT00000000. The version described in this paper is the first version, BIMT01000000. The raw reads were deposited in the SRA/DRA/ERA under the accession numbers DRA008301 and DRA008302. The strain is available from the Japan Collection of Microorganisms, RIKEN BioResource Research Center (Tsukuba, Ibaraki, Japan), under strain number JCM 33374.
